# Which cell death modality wins the contest for photodynamic therapy of cancer?

**DOI:** 10.1038/s41419-022-04851-4

**Published:** 2022-05-13

**Authors:** Tatiana Mishchenko, Irina Balalaeva, Anastasia Gorokhova, Maria Vedunova, Dmitri V. Krysko

**Affiliations:** 1 Institute of Biology and Biomedicine, National Research Lobachevsky State University of Nizhny Novgorod, Nizhny Novgorod, Russian Federation; 2 Cell Death Investigation and Therapy Laboratory, Department of Human Structure and Repair, Ghent University, Ghent, Belgium; 3 Cancer Research Institute Ghent, Ghent, Belgium; 4 Department of Pathophysiology, Sechenov First Moscow State Medical University (Sechenov University), Moscow, Russian Federation

**Keywords:** Cancer immunotherapy, Cell death and immune response

## Abstract

Photodynamic therapy (PDT) was discovered more than 100 years ago. Since then, many protocols and agents for PDT have been proposed for the treatment of several types of cancer. Traditionally, cell death induced by PDT was categorized into three types: apoptosis, cell death associated with autophagy, and necrosis. However, with the discovery of several other regulated cell death modalities in recent years, it has become clear that this is a rather simple understanding of the mechanisms of action of PDT. New observations revealed that cancer cells exposed to PDT can pass through various non-conventional cell death pathways, such as paraptosis, parthanatos, mitotic catastrophe, pyroptosis, necroptosis, and ferroptosis. Nowadays, immunogenic cell death (ICD) has become one of the most promising ways to eradicate tumor cells by activation of the T-cell adaptive immune response and induction of long-term immunological memory. ICD can be triggered by many anti-cancer treatment methods, including PDT. In this review, we critically discuss recent findings on the non-conventional cell death mechanisms triggered by PDT. Next, we emphasize the role and contribution of ICD in these PDT-induced non-conventional cell death modalities. Finally, we discuss the obstacles and propose several areas of research that will help to overcome these challenges and lead to the development of highly effective anti-cancer therapy based on PDT.

## Facts

- PDT is a widely used and useful tool in the treatment of multiple types of cancer, but its treatment modes still need to be adjusted to the needs of each patient according to the individual characteristics of a tumor.
- The efficacy of PDT depends largely on the activation of ICD that enables the immune system to engage in the combat against any remaining tumor cells and to generate long-lasting immunological memory.
- Many cell death modalities with immunogenic or non-immunogenic properties that can be induced by PDT have been recently discovered, and this list is still growing. They include mitotic catastrophe, paraptosis, pyroptosis, parthanatos, necroptosis and ferroptosis.
- To overcome tumor cell resistance to cell death, there is a need to induce different cell death types, including mixed cell death types, coupled with different combinations of PDT irradiation regimens and photosensitizers. This will provide novel and effective therapeutic strategies and will contribute to a so-called “ideal protocol” for PDT in cancer therapy.

## Open questions

- Which factors determine how to trigger of specific PDT-induced cancer cell death type?
- How can we overcome the resistance of cancer cells to certain types of death, and in particular apoptosis and necroptosis? Is the induction of mixed types of cell death a promising approach for overcoming such resistance?
- Is it worthwhile to induce ferroptosis by PDT during development of new approaches aimed at increasing the effectiveness of PDT against resistant and highly malignant tumors?
- An ideal protocol for PDT of cancer needs to be formulated.

## Introduction

The discovery of the main principles of photodynamic reactions more than 100 years ago [1–3] gave a powerful boost to research on the possibility of using photodynamic therapy (PDT) in various fields of medicine, including the treatment of tumors of different origins (Box 1).

**Fig. 1:**
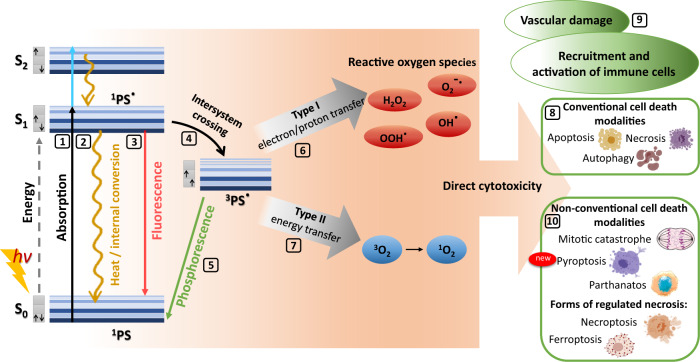
Mechanisms of photodynamic reactions and biological effects induced by photodynamic therapy. The photosensitizer (PS) accumulates in cancer cells, absorbs photons (*hv*) from a light source of appropriate wavelength, and is transformed to the short-lived excited singlet state (^1^PS•) [1] [11]. ^1^PS• can lose its energy by internal conversion into heat [2] or by emitting light (fluorescence) [3]. ^1^PS• could also be transformed into a long-lived excited triplet state (^3^PS•) *via* an intersystem crossing process [4]. Besides the ability of reversion to a singlet state (^1^PS) by emission of light (phosphorescence) [5], ^3^PS• can launch two kinds of reactions with adjacent molecules [6, 7]. The result of type I photochemical reaction [6] lies in ^3^PS• transferring an electron or a proton and formation of organic radicals. These radicals can interact with cellular oxygen to generate cytotoxic reactive oxygen species (ROS) (e.g., superoxide anion (O_2_^–^•)), hydroperoxide radical (HOO•), peroxides (H_2_O_2_, ROOH) and hydroxyl radical (HO•); this starts free radical chain reactions. The type II photochemical reaction [7] initiates triplet−triplet energy transfer of ^3^PS• to molecular oxygen, resulting in the formation of singlet oxygen (^1^O_2_), which is a powerful oxidizing agent [11]. Type I and type II photochemical reactions can occur simultaneously, and the ratio between them depends mainly on the photochemical and photophysical characteristics of the PS, and the concentrations of the substrate and cellular oxygen [11]. Type I and type II photochemical reactions can trigger different cell death mechanisms that are directly cytotoxic to the cancer cells. Traditionally, cell death induced by PDT was categorized into type I (apoptosis), type II (cell death associated with autophagy), and type III (necrosis) [8]. PDT also activates the recruitment and activation of immune cells and causes vascular damage [9]. However, in recent decades, several alternative cell death modalities that can be triggered by PDT have been identified [10]. These findings show that our knowledge of PDT of cancer has expanded.

PDT is a minimally invasive procedure that effectively kills tumor cells but has low toxicity for healthy tissues. The PDT procedure consists of several steps: systemic, local, or topical administration of a light-sensitive dye (photosensitizer; PS), its accumulation in tumor cells, and subsequent excitation by irradiation with visible light of appropriate wavelength (Fig. 1). The excited PS (a singlet state ^1^PS•) can undergo transformation into a long-lived excited triplet state (^3^PS•) *via* an intersystem crossing process that launches two kinds of photochemical reactions with adjacent molecules. In the type I photochemical reaction, ^3^PS• interacts directly with polyunsaturated fatty acids in cell membrane lipids, to which it transfers an electron or a proton to form organic radicals. Upon interaction with cellular oxygen, these radicals can generate cytotoxic reactive oxygen species (ROS), e.g., superoxide anion (O_2_^–^•), hydroperoxide radical (HOO•), peroxides (H_2_O_2_, ROOH) and hydroxyl radical (HO•), which launch free radical chain reactions. The type II photochemical reaction initiates triplet−triplet energy transfer of ^3^PS• to molecular oxygen, resulting in the formation of singlet oxygen (^1^O_2_), which serves as a powerful oxidizing agent. Type I and type II photochemical reactions can proceed simultaneously, and the ratio between them depends mainly on the photochemical and photophysical characteristics of the PS and the concentrations of substrate and cellular oxygen. Type I and type II photochemical reactions trigger different cell death mechanisms that are directly cytotoxic to cancer cells and lead to tumor tissue destruction (Fig. 1). PDT also has a direct effect on the tumor vasculature and causes shutdown of vessels, which deprives the tumor of oxygen and nutrients [4–7].

**Fig. 2:**
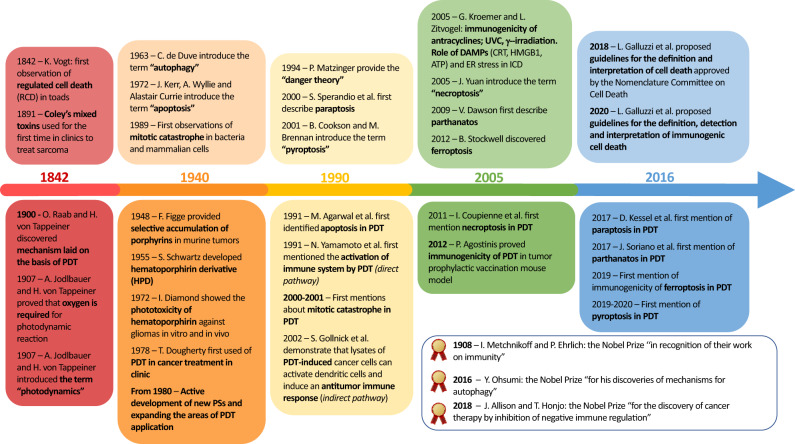
A timeline of cell death modalities and PDT.

Besides the “*conventional”* cell death pathways of apoptosis, necrosis and autophagy (Box 2), other “*non-conventional*” cell death modalities that can be triggered by photodynamic reactions have been revealed in recent decades, such as regulated forms of necrosis, including necroptosis, ferroptosis, pyroptosis, parthanatos and mitotic catastrophe (Fig. 2). These findings have provided new insights into the PDT-induced death signaling pathways. Moreover, the emergence of new methodological approaches for determining the details of cell death mechanisms makes it imperative to reexamine our findings on cancer PDT, and to revise our knowledge where appropriate.

The fate of cancer cells exposed to PDT largely depends on the tumor’s origin, the photophysical and photochemical properties of the PS [5, 8], its tissue distribution [9], its uptake by cancer cells and its subcellular localization [8, 10, 11], and the irradiation dose [12, 13]. Notably, the photodynamic reactions can trigger one or several cell death modalities simultaneously [14–17], which directly affects the therapeutic outcome of PDT. Though PDT has entered the clinic for primary and adjuvant anticancer treatment (e.g., cancers of the head and neck, skin, brain, digestive system, urinary system, lung, and mesothelioma), the mechanisms of the photodynamic effects on tumor cells have not been fully established yet. The current direction in PDT research is aimed at making PDT a more appealing therapeutic option, and particularly to explore the possibility of photodynamic induction of cell death modalities that can overcome tumor resistance to apoptosis.

William B. Coley discovered the positive effect of a “vaccine” based on a mixture of killed erysipelas-causing bacteria (nowadays called Coley’s toxins) in patients with non-operable sarcomas in the 19th century [18, 19], but it was only in 1994 that Polly Matzinger proposed the “danger theory” [20, 21]. Later, the concept of immunogenic cell death (ICD) proposed by Guido Kroemer’s group in 2005 [22] explained the detrimental effect of the immune system to the success of antitumor therapy. Since the late 1900s, PDT has attracted attention not only as an independent method for anti-tumor therapy, but also as a strategy for activating the immune response [11, 23–25]. Cells undergoing ICD gain the ability to emit several immuno-stimulatory molecules called damage-associated molecular patterns (DAMPs). These include ATP, calreticulin (CRT), high-mobility group Box 1 (HMGB1), heat shock proteins (HSPs) 70 and 90, and cytokines/chemokines promoting the recruitment and maturation of antigen-presenting cells by cancer cells that are in the process of dying [26–28]. This process culminates in the cross-presentation of antigenic peptides on major histocompatibility complex class I (MHC I) molecules to CD8^+^ T cells of the adaptive immune system [29], a major driving force of effective control of tumors and long-term anti-cancer immunity.

Following the principles of cancer immunotherapy, an ideal PDT protocol should successfully destroy the primary tumor while activating the immunogenic cell death pathway. This allows the activation of the immune system to identify and destroy residual tumor cells, including those in distant metastases. Notably, PDT can induce different immunogenic cell death modalities, e.g., apoptosis [11], necroptosis [11, 30, 31], and ferroptosis [14, 32, 33] as well as death pathways with unknown immunogenic properties [34, 35]. This suggests that different cell death modalities can boost PDT efficacy whereas others may compromise the therapeutic outcome.

In this review, we discuss non-conventional cell death modalities that can be induced by PDT. We focus on mitotic catastrophe, paraptosis, pyroptosis, parthanatos, necroptosis, and ferroptosis, and we assess their relevance in anticancer therapy. Next, we emphasize the role and contribution of ICD in these PDT-induced non-conventional cell death modalities. Finally, we discuss the prospects for developing strategies to enhance PDT efficacy in cancer treatment.

Box 1 Anti-cancer PDT in the clinic: the current state and new trends After a century, PDT has taken its rightful place among the powerful cancer treatment modalities [201, 202]. PDT involves topical or systemic administration of photoactive dye (photosensitizer, PS), followed by irradiation of the lesion with visible or NIR light to induce cytotoxic photochemical reactions.

Several groups of PSs have been clinically approved for anti-cancer PDT, with most of them containing porphyrin compounds as an active ingredient [203]. These are derivatives of hematoporphyrin, chlorin, bacteriochlorin, phthalocyanine, and bacteriopheophorbide. The treatment based on topical or oral administration of 5-aminolevulinic acid (5-ALA) and its methyl ester, which are precursor drugs leading to accumulation of endogenous protoporphyrin IX in cancer tissue, should be separately noted. The long list of non-porphyrin and porphyrin candidate PSs are undergoing (pre)clinical trials [204, 205].

Among the most important advantages of PDT is low systemic toxicity because the cytotoxic effect of the PS in the absence of light irradiation is several orders of magnitude lower than in the irradiated cancer tissue. But a current limitation is the mode of light delivery, which is currently restricts treatment to superficial tumors or tumors accessible with an endoscope: non-melanoma skin cancer [206], retina and ocular malignancies [207], cancer of the digestive tract [208], urinary system malignancies [209, 210], tracheobronchial malignancies [211], and cervical cancer [212]. Also, PDT was shown to be effective in improving patient prognosis when used after surgical resection of glioblastoma [213, 214]. Large solid tumors and tumors located deep in brain and parenchymal organs require interstitial PDT (IPDT) [215]. This approach involves delivery of light by optic fibers inserted via needles or catheters. IPDT has proven to be successful in the treatment of primary and recurrent prostate cancer [216], breast cancer [217], glioma [218, 219], head and neck tumors [220], and pancreatic cancer [221]. The progress in light delivery systems implies expansion of the list of tumor localizations that can become accessible to PDT. An elegant technical solution is the use of wireless optoelectronic devices fixed in the body to provide local continuous low-power irradiation of a tissue for up to several days, an approach named metronomic PDT (mPDT) [222, 223].

Strictly speaking, artificial light sources are not required for PDT. The recently developed daylight PDT (dPDT) has become a powerful tool for treatment of skin cancer and precancerous lesions [224, 225]. In this treatment modality, topical application of 5-ALA or its derivatives is followed by dosimetry-controlled sunlight exposure. The procedure is cost effective, avoids pain, and minimizes inflammatory side effects compared with conventional PDT protocols.

PDT can be applied as a primary therapy in some clinical situations, but it has also proven to be a potent adjuvant anticancer treatment when combined with surgery, chemotherapy or radiotherapy. Since demonstration of the induction of ICD by PDT and its promotion of anticancer immune responses in the last decade [11, 226], intensive studies have aimed at maximizing the effect by combining PDT with immuno-therapeutics, and the first steps to translating this approach to the clinic have been reported [227].

Box 2 Conventional and non-conventional cell death modalities in PDT – which is which? Over several decades, cell death induced by PDT was conventionally categorized into three types, the so-called “three classical pillars” of cell death: type I (apoptosis), type II (autophagy), and type III (necrosis) [6, 228]. When PDT is used according to stringent regimens (e.g., high concentrations of PS or strong irradiation doses), cancer cells undergo rapid and non-regulated necrotic cell death (also termed accidental necrosis), which is known to be non-immunogenic [14, 15, 32, 134]. In contrast, the optimized PDT procedure guides cancer cells through regulated cell death pathways. Molecular mechanisms of PDT-induced apoptosis and autophagy, which can simultaneously induce pro-survival as well as cytotoxicity effects, are the most extensively studied pathways of photokilling and are well described in previous reviews [6, 187, 228, 229].

The striking progress made in the cell death field in recent years greatly expanded our knowledge of the molecular mechanisms of the cell death modalities and led to the discovery of new regulated forms of cell death, such as necroptosis and ferroptosis (Fig. 2). The *non-conventional* forms of cell death generated by PDT have different morphological, biochemical, genetic, and functional properties from the well-known “three pillars” of cell death. The guidelines for definition and interpretation of cell death approved by the Nomenclature Committee on Cell Death in 2018 describe more than 10 cell death modalities [38]. At the same time, there is an unceasing stream of studies that describe new forms of cell death differing in their characteristics from the currently recognized forms, and for which a reliable set of biomarkers is being developed. Therefore, in this review we focus on non-conventional cell death modalities, such as mitotic catastrophe, paraptosis, pyroptosis, parthanatos, necroptosis, and ferroptosis, and on the assessment of their relevance to anticancer therapy.

## Mitotic catastrophe

### A brief overview of mitotic catastrophe

Mitotic catastrophe (MC, Fig. 2) is a cell death modality triggered by aberrant mitosis leading to chromosome mis-segregation and failed cell division [36, 37]. In the Nomenclature Committee on Cell Death (2018), MC is considered as a regulated onco-suppressive mechanism that impedes the proliferation and/or survival of cells that cannot complete mitosis due to extensive DNA damage, defective mitotic machinery, and/or failure of mitotic checkpoints [38]. Morphologically, MC is characterized by specific nuclear changes, including multinucleation and macronucleation presumably related to chromosomal mis-segregation, as well as micronucleation apparently related to the persistence of lagging or acentric chromosomes. The specific molecular mechanisms associated with MC development are not fully understood, but inhibition of this cell death pathway by p53 has been reported [36, 39, 40].

MC triggers a delayed cell death response that culminates in morphological features closely resembling apoptotic or necrotic cell death or an irreversible cell cycle arrest termed senescence. MC is closely linked with sub-lethality or delayed lethality of different pharmacological and/or physical stressors, including ionizing radiation [41], hyperthermia [42], and chemotherapeutics [43], that directly affect DNA integrity or disrupt/block mitotic spindles, and thereby block mitosis progression at metaphase. Cytoskeletal components are considered the most sensitive cellular components to MC. For instance, chemotherapeutic agents that can induce MC are used to impede microtubule dynamics. These agents include vinca alkaloids (e.g., vincristine, vinorelbine), taxanes (e.g., paclitaxel, docetaxel), nocodazole [44, 45], actin blockers, e.g. cytochalasins, inhibition of cytokinesis, and proteins involved in cell cycle checkpoints (e.g., auroraA/B, polo-like kinase-1, and mitotic arrest deficient 2) [36, 46]. Interestingly, MC may be accompanied by the appearance of ICD hallmarks. Microtubular poisons and inhibitors of the actin cytoskeleton can stimulate eIF2α phosphorylation, which leads to exposure of clareticulin (a key DAMP) on the plasma membrane, with further induction of anti-tumor immune responses [46, 47].

### Mitotic catastrophe in photodynamic cancer treatment

PDT can trigger MC, and one of the first observations of this was in studies focused on PDT based on second-generation PSs [11] (Table 1), many of which are currently used in the clinic. Therefore, deeper study of the molecular mechanisms of cell death in PDT induced by these PSs will provide new insight into the complexity and implications for cancer therapy and for timely modification of the treatment strategy. In contrast to the action of chemotherapeutic drugs, PDT-induced of MC *via* cell cycle block is not always accompanied by direct damage of cytoskeletal structures. The PSs triggering MC may have different cellular localizations (Table 1). Since the singlet oxygen can travel only about 10–20 nm, the most damaged location during PDT is at the site of the PS. Therefore, it can be assumed that ROS production during PDT primarily affects sites of PS localization that are in close proximity to microtubules; alternatively, the microtubule damage can be mediated by the secondary formation of free radicals.

**Table 1:** PDT based on second generation photosensitizers can induce mitotic catastrophe in cancer cells.

Photosensitizer	Type of cancer cells and subcellular localization	Signs of mitotic catastrophe	Refs
Methyl-aminolevulinate (MAL) (precursor for endogenous Protoporphyrin IX)	HeLa human cervical adenocarcinoma: plasma membrane and lysosomes, partially in the ER and Golgi apparatus	Mitotic arrest at metaphase-anaphase transition, multipolar spindles, disorganized chromosomes, dispersion of centromeres, and alterations in aurora kinase proteins	[55]
α,β,χ,δ porphyrin-Tetrakis (1-methylpyridinium-4-yl) p-Toluenesulfonate porphyrin (TMPyP)	HeLa human cervical adenocarcinoma: lysosomes [192]; G361 human melanoma – presumably in lysosomes	Increasing of mitotic index early after PDT, cell cycle arrest, microtubule disorganization of interphase cells, aberrant mitosis	[48]
TMPyP4	A549 human lung cancer: localization not analyzed	Alteration of expression of particular genes and/or the interference with DNA replication	[193]
5,10,15-tris(pentafluorophenyl)corrole (TPFC) and its β-cyclodextrin conjugates (β-CD1, β-CD2)	HeLa human cervical adenocarcinoma: lysosomes and Golgi apparatus	Altered configurations of the mitotic spindle, presence of extrapoles, chromosome arrangements	[194]
(5-(4′-(2″-dicarboxymethylamino) acetamidophenyl)-10, 15, 20 triphenylporphyrin)	A549 human lung cancer: mitochondria and lysosomes	S cell cycle arrest	[57]
5-5-(4-N,N-diacetoxylphenyl-10, 15, 20-tetraphenylporphyrin)	MCF-7 luminal A type breast cancer: mitochondria and lysosomes	G0/G1 cell cycle arrest (0.5 h after PDT)	[195]
Indocyanine green	B16F10 murine melanoma: localization not analyzed	G0/G1 cell cycle arrest (immediately after photodynamic hyperthermal therapy)	[196]
Photocyanine	HepG2 human hepatocellular carcinoma: lysosome and mitochondria	G2/M cell cycle arrest	[197]
Asymmetric glycophthalocyanine GPh3	HeLa human cervical adenocarcinoma: Golgi apparatus, partially lysosomes, the cell membrane, and diffused into the cytoplasm	G2/M cell cycle arrest leading to formation of multiple spindle poles. The effects were partially negated by the pancaspase inhibitor Z-VAD-FMK	[198]
Tetra-α-(4-carboxyphenoxy) phthalocyanine zinc (TαPcZn)	Bel-7402 human hepatocellular carcinoma: plasma membrane and nuclear membrane	S cell cycle arrest with concomitant downregulation of Bcl-2 and Fas	[199]
Zn(II)-phthalocyanine (ZnPc)	HeLa human cervical adenocarcinoma: single wide area near the nucleus (Golgi apparatus)	Metaphase blockage, aneuploidy, presence of abnormal mitotic spindles (> supernumerary poles)	[200]
Hypericin	HeLa human cervical adenocarcinoma: endoplasmic reticulum and Golgi apparatus, co-localization with α-tubulin	G2/M cell cycle arrest, aberrant mitotic spindles	[53]

The damage or disorganization of microtubules induced by PDT could be related to the arrest of different phases of the cell cycle (Table 1). Interestingly, mitotic block could develop not only due to PDT but could also be caused by the PS without photoinduction. This assumption is based on findings describing a significant increase in the mitotic index both immediately after PDT and during incubation of tumor cells with the PS in the dark [48]. Destabilization of DNA and protein interactions in the nucleosomes can lead to alterations in DNA structure and/or gene expression, thus increasing the sensitivity to the photoreaction [49–51]. Some porphyrins can bind tubulins and inhibit their polymerization during PDT or without it [52]. The damage to microtubules and the triggering of alterations in the mitotic spindle are also typical of hypericin-based PDT [53].

Disruptions of microtubule organization interact closely with centrosomal proteins such as AuroraA, ninein, TOG, and TACC3 [54]. Depletion of these proteins enhances aberrant spindle formation and results in apoptotic-like cell death. Slight effects on spindle morphology also lead to depletion of microtubular nucleator γ-tubulin [54, 55]. Atomic force microscopy showed that PDT based on the chlorin-based photosensitizer DH-II-24 disrupts the cytoskeleton in J82 bladder cancer cells and further leading to activation of apoptosis [56]. Disruption of the cytoskeletal structure can be associated with proteolytic cleavage of proteins in direct contact with the cytoskeleton when the mitochondria and lysosomes are damaged by PDT-generated ROS [57, 58].

However, it has been shown that tumor cells can bypass mitotic blockade during PDT, which increases the risk of aneuploidy and genomic instability, and consequently the tumors become more aggressive and resistant to therapy [37, 59]. When PDT acts on cytoskeletal components, microtubule damage may be transient [60, 61]. Thus, although some cells with damaged microtubules will most likely die, some are partially resistant to the PDT-induced disruption of microtubules, and quite likely to PDT in general. Such variable responses are supposed to occur when the lethality of PDT is inadequate or delayed [48]. It is therefore assumed that the mechanism of PDT-induced depolymerization of microtubules is reversible [48] or cells with damaged microtubules do not survive the PDT treatment [62]. The reversibility of microtubule damage and resistance to microtubular recovery could be overcome through the different pathways leading to cell death by adjusting of PS concentration and the irradiation dose. Therefore, though more detailed morphological, biochemical and genetic studies of mitotic catastrophe are very labor-intensive, they are needed as a part of the investigation of the mechanisms of PDT.

## Paraptosis

The Nomenclature Committee on Cell Death had not approved the term “paraptosis” by 2018 [38], presumably because there were no reliable biomarkers for it other than routine examination of cellular morphology. Paraptosis was first described by Sperandio et al. in 2000 [63] as characterized by cytoplasmic vacuolation and mitochondrial and/or ER swelling (Fig. 2). Paraptosis is associated with protein synthesis and thus can be blocked by the translation inhibitor cycloheximide. In contrast to apoptosis, paraptosis does not exhibit chromatin condensation and cell fragmentation, and it is independent of сaspase activation. Paraptosis can be initiated during neuronal development by mutations in the insulin-like growth factor domain [63]. On the other hand, paraptosis is accompanied by alteration of Ca^2+^ and redox homeostasis, it is dependent on mitogen-activated protein kinase (MAPK) family members, and it can be inhibited by the multifunctional adapter protein AIP-1/Alix [64, 65]. Moreover, several studies on PDT demonstrated both MAPK-dependent and MAPK-independent pathways leading to paraptosis after photodamage of the endoplasmic reticulum (ER) [66].

### Paraptosis and PDT

In contrast to autophagy, paraptosis is accompanied by the formation of vacuoles bound by a single rather than a double membrane. This morphological feature was observed by D. Kessel’s group in PDT based on hypericin and a benzoporphyrin derivative (BPD, Verteporfin) in human ovarian carcinoma cells (OVCAR-5) [67]. Notably, vacuole formation could be suppressed by MAPK antagonists or inhibition of new protein synthesis when low doses of Verteporfin-PDT were used. However, for clinically relevant doses, the signaling pathway to paraptosis was mostly independent of these factors [67]. It is noteworthy that stringent PDT regimens, which increase cell mortality above LD_90_, cause crosslinking of ER proteins followed by impairment of the paraptotic response, which is assumed to be related to limitation of the mobility of ER proteins and results in an inability to form typical paraptotic vacuoles [35, 66].

### The role of PS localization in paraptosis induction

It has been suggested that paraptosis seems to be a cell death pathway functional in cells with an impaired apoptotic program and is seemingly unaffected by autophagy [66]. Because paraptosis is usually associated with the appearance of misfolded proteins in the ER, it is reasonable to assume that the PS used in PDT should be targeted primarily to the ER. Activation of paraptosis was shown in different cancer cell lines in PDT based on PSs localized predominantly in the ER, such as pyropheophorbide HPPH [67], hypericin [68, 69] and verteporfin [70–72]. The signs of paraptosis were observed in human-derived non-small-cell lung cancer A549 cells and mouse hepatoma 1c1c7 cells four hours after PDT based on using a LD_90_ dose of m-tetra(hydroxyphenyl) chlorin (m-THPC) [73]. In contrast, the paraptotic processes were absent in PDT based on chlorin NPe6, which localizes in lysosomes [67]. However, occasional publications show that paraptosis was induced by photodamage when the PS was localized elsewhere. For instance, cytoplasmic vacuoles of ER origin were observed in PDT based on [Ru(bipy)2-dppz-7-methoxy][PF6]2 (Ru65), which localizes predominantly in the nucleus [74].

### Paraptosis in PDT – what is next?

Recent studies have shown that various thiol-containing antioxidants, such as GSH, can influence the rate of paraptosis and block it [75]. Therefore, it has been suggested that decreasing the high levels of GSH expressed in tumor cells will facilitate the development of paraptosis and enable its assessment as an anti-cancer therapy.

Hypoxia in the tumor microenvironment reduces the effectiveness of PDT significantly. To overcome this, Han et al. recently constructed a nanoplatform (GC@MCS NPs) [76] based on hypoxia-responsive hyaluronic acid-nitroimidazole (HA-NI), MnO_2_ nanoparticles (MnO_2_ NPs) as oxygen modulators, and poly (l-glutamic acid) derivatives (γ-PFGA) as cores to deliver gambogic acid (GA, paraptosis inducer), and the PS Chlorin e6 (Ce6). Chemo-PDT based on GC@MCS NPs provided efficient tumor oxygenation due to MnO_2_ NPs, whereas GA-loaded γ-PFGA-assisted deep penetration of Ce6 with subsequent cell death of tumor 4T1 cells in vitro and in vivo [76]. All these processes were accompanied with gradual aggregation and fusion around vacuoles of ER and mitochondrial origin, which suggests the activation of paraptosis. At the same time, PDT-generated ROS contributed to potentiation of GA-induced paraptosis by decreasing the intracellular level of GSH, which can block the effects of paraptosis. On the other hand, the effect on the GSH-redox system during PDT can activate another cell death pathway, e.g., ferroptosis [77, 78]. Thus, elucidating the mechanisms of cancer cell death induced by GC@MCS NPs-PDT will open new prospects for research.

Moreover, paraptosis can be activated through the large potassium BK channels, which control cellular swelling and vacuolization of cancer cells, accompanied by the appearance of the ICD hallmarks. Prolonged BK channel activation can result in overexpression of heat shock proteins (Hsp) 60, 70, 90 and gp96, release of one of the key DAMPs (HMGB1), and enhancement of the tumor immunogenicity of rat glioma T9 cells [79]. However, the release of HMGB1 in verteporfin-based PDT with a LD _90_ dose was not significant; this questions the use of HMGB1 measurement as a marker of PDT-induced paraptosis [80]. Although the morphological signs of paraptosis are observed, the absence of ICD hallmarks can be considered as a non-classical response to high doses of PDT.

Thus, the determinants and consequences of paraptosis are not fully understood. Further research will clarify whether paraptosis can be considered as an effective PDT strategy for killing cancer cells and activating the immune system.

## Pyroptosis

### A brief overview of pyroptosis

Pyroptosis is an inflammatory form of regulated cell death first observed in macrophages infected with *Shigella flexneri* [81] and later induced by *Salmonella typhimurium* [82]. Pyroptosis has also been found in other microbial infections, heart attack, stroke, and tumorigenesis [83, 84]. This cell death modality has different names: pyronecrosis, gasdermin-dependent cell death, and caspase 1-dependent cell death. However, the term “pyroptosis” (Greek root “pyro”, which means fire or fever, and “ptosis”, which means falling), proposed by B. Cookson and M. Brennan in 2001, is now officially approved by the Nomenclature Committee on Cell Death (Fig. 2) [38, 85]. Pyroptosis can have the morphological features of both apoptosis and necrosis. In the early stage, pyroptosis is accompanied by apoptosis-like DNA fragmentation and chromatin condensation, followed by the appearance of necrosis-like morphological changes causing the release of inflammation molecules. The specific morphological and biochemical characteristics of pyroptosis make it easy to distinguish from other cell death modalities. Pyroptosis is associated with the activation of one or more caspases, including caspase 1, caspase 3, murine caspase 11, and human caspase 4 and caspase 5 in the inflammasome (e.g., NLRP3 [86], NLRC4 [87], AIM2 [88, 89], Pyrin [90]), a multiprotein complex comprising sensors of pathogens and effector molecules.

In the canonical, caspase-1 inflammasome pathway, pyroptosis involves recognition of DAMPs or PAMPs by pattern recognition receptors (e.g., ALRs, TLRs, NLRs), and this leads to the formation of specific inflammasomes. In this scenario, caspase-1 triggers the protein complex, which contains the inflammasome sensor, adaptor protein apoptosis-associated speck like proteins (ASC), and caspase activation and recruitment domain (CARD) of ASC, which play an important role [91]. At the same time, active caspase 1 induces the maturation and secretion of the pro-inflammatory cytokines interleukin 1β (IL-1β) and IL-18, leading to inflammation. Moreover, caspase activation often catalyzes the proteolytic cleavage of gasdermin protein family, primarily gasdermin D (GSDMD), which oligomerizes in membranes and forms non-selective pores that rapidly permeabilize the plasma membrane [92, 93]. These changes increase water influx and disturb the ionic gradient, cause cell swelling and membrane rupture, and culminate in cell lysis and release of inflammatory intracellular contents [94].

In the non-canonical pathway, caspase-4/5 (human) and caspase 11 (mice) recognize and bind to cytosolic lipopolysaccharides in invading Gram-negative bacteria, leading to cleavage of gasdermin-D and activation of pro-IL-18 and pro-IL-1β [95]. The caspase-3/GSDME-induced pathway can launch pyroptosis [96].

### Pyroptosis in cancer treatment: pros and cons

The relationship between pyroptosis and cancer is complex, and the effects of pyroptosis largely depend on the tumor’s origin and the genetic background [97]. On the one hand, pyroptosis can be considered as a protumorigenic mechanism of cell death because the inflammatory cytokines produced as a result of pyroptosis provide a suitable microenvironment for tumor progression and metastasis [98, 99]. On the other hand, several chemotherapeutic drugs, such as paclitaxel [100], cisplatin [100], and simvastatin [101], can induce pyroptosis, which contributes to the development of robust antitumor activity [96]. Interestingly, it has been shown that pyroptosis exhibits several ICD hallmarks, such as the release of DAMPs (e.g., HMGB1 [102, 103], ATP [104], mtDNA [105]) and DAMP-like molecules, e.g., ASC specks [106–108]. Therefore, it can also be considered an immunogenic cell death modality that could significantly increase the efficacy of anti-cancer therapy.

In a recent elegant study, peroxydisulfate nanoparticles Na_2_S_2_O_8_ (PNSO NPs) highly cytotoxic against murine breast cancer 4T1 cells were developed [109]. The anti-tumor effects were mediated by the generation of reactive species, including the recently discovered and highly toxic ^•^SO_4_−, and also by alterations in cellular osmolarity facilitating rapid caspase-1-related pyroptosis [109]. At the same time, the cancer cell death was characterized by pronounced immunogenic properties in vitro and activation of T cell antitumor immunity (CD8^+^ and CD4^+^ T cells) in a murine therapeutic model in vivo. In combination with CTLA4-blocking immunotherapy, it was also characterized by the induction of a systemic antitumor immune response; this approach was effective in combating lung metastasis and recurrence [109].

### Role of PDT in induction of pyroptosis

In a different approach to PSs, novel membrane-anchored PSs with aggregation-induced emission characteristics (1,1,2,2-tetraphenylethene-benzo[c] [1, 2, 5]) and thiadiazole-2-(diphenyl methylene) malononitrile (TBD) have recently been synthesized [110]. PDT with TBD-3C induced both apoptosis and pyroptosis (but predominantly pyroptosis) in murine breast cancer 4T1, human adenocarcinoma, HeLa cells, and rat glioma C6 cells [110]. In addition to the typical morphological changes (e.g., formation of pyroptotic bubbles), cancer cell death was accompanied by ROS-induced activation of caspase-1 with subsequent GSDMD cleavage, and release of the inflammatory cytokines IL-1β and IL-18 [110]. It has also been shown that curcumin-loaded poly(L-lactide-co-glycolide) microbubbles in sono-PDT induce pyroptosis and apoptosis in human liver cancer HepG2 cells [111].

Another study examined the mechanisms of PDT based on 5-ALA, protoporphyrin IX dimethyl ester and chlorin e6 in esophageal squamous carcinoma cells. In that study, pyruvate kinase M2 (PKM2), which catalyzes the last step of glycolysis and is essential for the Warburg effect, was found to play an important role in PDT-induced pyroptosis [112]. PDT downregulates PKM2 expression and consequently activates caspase-8 and caspase-3, eventually resulting in the release of N-GSDME and leading to pyroptosis induction. At the same time, the effect of PDT was not reflected in the levels of GSDMD and caspase-1 [112]. It should be noted that the overexpression of PKM2 blocked pyroptosis in PDT treatment. The novel PKM2/caspase-3/GSDME pyroptosis pathway raises the question of whether PKM2 is a promising and generally relevant target for the use of pyroptosis in PDT, and also suggests a new strategy and theoretical basis for improving PDT efficacy.

It is important to stress that pyroptosis in the context of PDT is still largely ignored by scientists. ROS production induced by chemotherapeutic drugs can act as a driving force in pyroptotic cancer cell death [113]. As PDT triggers the generation of ROS (Fig. 1), it should receive much more attention by exploring its potency as an alternative pathway for effectively killing cancer cells. PDT is accompanied by local acute inflammation closely linked with the production of pro-inflammatory cytokines that upregulate the immune system response by attracting host leukocytes into the tumor and increasing antigen presentation. Local inflammation can switch to a systematic (generalized) response that can affect the long-term PDT outcomes. However, it should be noted that neutralization of IL-1β by inhibition of caspase-1 in the inflammasome can reduce the cure rate of PDT-treated tumors [114]. This means that cytokine networks upregulating the immune system in PDT should be remain balanced. This balance might be achieved by controlling the dose of light used in PDT. For instance, PDT based on a low dose of pyropheophorbide HPPH is not very effective against cutaneous squamous cell carcinoma cells, but the extent and nature of PDT-induced acute local inflammation may affect the long-term therapeutic outcome [115]. Studies are needed to better understand the role of the balance of cytokines in PDT-induced pyroptosis in cancer therapy. This is indeed an intriguing direction for future research.

## Parthanatos

### A brief overview of parthanatos

Parthanatos is a regulated form of cell death that specifically depends on the hyperactivation of the DNA damage response machinery, namely Poly (ADP-ribose) polymerase (PARP1), and it is independent of caspases (Fig. 2) [38, 116]. Activation of PARP1 can be initiated by different stimuli that damage DNA, including ultraviolet irradiation, alkylating agents and ROS generation [117–119]. PARP1 overactivation causes the accumulation of PAR polymers and cellular NAD^+^ and depletion of ATP, resulting in energetic collapse. This cell death process is accompanied by mitochondrial depolarization, which contributes to the release from mitochondria of the truncated form of apoptosis-inducing factor (AIF). Subsequent interaction of AIF with macrophage migration inhibitory factor (MIF) results in the formation of the AIF/MIF complex. This complex enters the nucleus, where it induces DNA fragmentation, triggering cell death [120–123]. Notably, PARP1 inhibitors (e.g., BYK204165, AG-14361, iniparib, 3-aminobenzamide and olaparib) or complete depletion of PARP1 block parthanatos, but caspase inhibitors do not [123–126]. Depending on various conditions, PARP1 can be either tumor-suppressive or tumor-stimulatory [127]. Thus, this controversial dual role of parthantos in cancer therapy should stimulate further research to elucidate the mechanisms of the parthanatos pathway and explore the safety and potential clinical outcomes and of using parthanatos-associated agents in anticancer therapy.

### Parthanatos in PDT

The only evidence available for PDT-induced parthanatos was provided by Soriano et al. [34]. That study compared the responses human breast epithelial cell lines (SKBR-3) and non-tumoral epithelial cells (MCF-10A) to PDT. The authors found that PDT based on *meso*-tetrakis (4-carboxyphenyl) porphyrin sodium salt (Na-H2TCPP) and its zinc derivative, Na-ZnTCPP, induce the activation of the apoptotic and necrotic pathways in SKBR-3 cancer cells [34]. In contrast, PDT-induced death in non-tumoral epithelial cells (MCF-10A) was characterized by a necrotic pathway combined with the characteristic features of parthanatos. In this cell population, PDT induced the formation of spotted nuclei and was accompanied by translocation of AIF from the mitochondria to the nucleus. Moreover, inhibition of PARP with 3-aminobenzamide increased the viability of MCF-10A cells in a dose-dependent manner, but it had no significant effect on the viability of SKBR-3 cells. It has been suggested that parthanatos seems to be mediated by DNA damage and PARP overactivation induced by a local increase in ROS during PDT, and by localization of the PS in the nucleus. Since PARP inhibition does not affect the efficiency of tumor cell death in PDT but significantly increases the survival of normal cells, it is reasonable to consider blocking parthanatos as a useful tool for development of treatments with less side effects for healthy tissues. It is important to understand the mechanisms of parthanatos induction by PDT in more detail. An intriguing question is whether parthanatos has immunogenic properties.

## Necroptosis

### A brief overview of necroptosis

The term *necroptosis* was coined by J. Yuan in 2005 for a nonapoptotic form of programmed necrotic cell death (Fig. 2) [128]. Necroptosis is well characterized and rightfully occupies a place in the Nomenclature on Cell Death [38]. Necroptosis can be triggered by multiple stimuli, including surface-associated death receptors, e.g., tumor necrosis factor receptor 1 (TNFR1), DR4/5 and FAS receptor, by pattern-recognition receptors such as Toll-like receptor 3 (TLR3), TLR4, and Z-DNA binding protein 1 (ZBP1), and by other stimuli that are well described in previous reviews [129, 130]. Morphologically, necroptosis closely resembles necrosis. The necroptotic process is characterized by cell swelling, moderate chromatin condensation, and rapid plasma membrane permeabilization with subsequent release of cellular content into the extracellular space. But signs of apoptotic cell death such as nuclear fragmentation, internucleosomal DNA cleavage and caspase activation are absent. According to a recent nano-topographical analysis in vitro, dying necroptotic cells do not shrink as in apoptosis, but swell and detach, with the formation of nanoscale pores (>200 nm) in the membrane [131]. The necroptotic process is characterized by a gradual decrease in cellular elasticity, so in the early stages of necroptosis the cytoskeletal structure remains intact [131]. Induction of necroptosis critically depends on the activity of receptor-interacting protein kinase-1 (RIPK1), RIPK-3, and mixed lineage kinase domain-like protein (MLKL).

Necroptosis can be blocked by RIPK1 inhibitors (e.g., necrostatin-1s), RIPK3 inhibitors (e.g., GSK’872) and MLKL (e.g., necrosulfonamide for human cells only). The absence of caspase activity in necroptosis makes it insensitive to pan-caspase blockers, e.g., zVAD-fmk, which prevents apoptosis in many different cell types. zVAD-fmk can sensitize cells to necroptosis by inhibiting the activity of caspase-8 [132, 133]. Activation of necroptosis followed by development of an anti-tumor immune response effectively protects against tumor growth in the presence of treatment with chemotherapeutic drugs, radiotherapy, anticancer vaccines or oncolytic viruses [30, 31, 134, 135]. Necroptosis is associated with the emission of several DAMPs (e.g., HMGB1 and ATP) and cytokines/chemokines [30, 31, 134]. It is noteworthy that the activation of the necroptotic cell death pathway can have a dual role in tumorigenesis [136]. On the one hand, massive release of HMGB1 and ATP or cytokines/chemokines during necroptosis could enhance the pro-inflammatory effect and induce attraction of myeloid-derived suppressor cells and/or tumor-associated macrophages, which eventually leads to tumor-associated immune suppression [136–138]. Moreover, cytokines released by necroptotic cancer cells can promote angiogenesis and cancer cell proliferation, thereby contributing to further tumor progression and metastasis. On the other hand, necroptosis is an ICD modality and can be considered as an alternative pathway for overcoming the apoptosis resistance of cancer cells to treatment, while promoting the activation of T-cell antitumor immune responses by emission of DAMPs, which act as adjuvants [30, 31, 134, 135]. In addition, unlike apoptosis, necroptosis may exhibit more pronounced antigenicity that provokes an antitumor immunogenic response directed toward not only endogenous tumor-associated antigens (e.g. AH1), but also to a mixture of neoepitopes [30].

### Necroptosis and PDT

One of the first reports on the induction of necroptosis by PDT was by Coupienne et al. [139], who showed that the production of singlet oxygen in PDT based on 5-aminolevulinic acid (5-ALA) is the cause of the activation of a RIPK-3-dependent cell death (necroptosis) in human glioblastoma LN18 cells. In that study, it was shown that RIPK1 and RIPK3 aggregate in an atypical necrosome complex lacking caspase-8 and FADD.

The tumor type and the concentration of the PS are important factors determining the possibility of necroptosis activation by PDT. For instance, in vitro and in vivo studies of the antitumor effect of hiporfin-based PDT in several cell lines of osteosarcoma (DLM-8, 143B and HOS) showed that cancer cells mainly underwent apoptosis [140]. However, the use of a low concentration of PS in human osteosarcoma 143B and HOS cell lines can also activate autophagy, which serves as a protective mechanism. What is more interesting is that hiporfin-PDT-driven cell death in murine osteosarcoma DLM-8 cells was accompanied by increased expression of RIPK1 and could be blocked by necrostatin-1, which points to the necroptotic pathway [140]. Moreover, application of a low concentration of talaporfin sodium (mono-L-aspartyl chlorine e6, NPe6) activates necroptosis in PDT treatment of human glioblastoma T98G cells, whereas the use of a high concentration of the PS induces non-necroptotic necrosis [141]. Low doses of N‐TiO_2_ nanoparticles stimulate a nontoxic autophagy flux response in human melanoma A375 cells, but the elevated ROS generation during photoactivation blocks autophagy and results in a switch of cell death to RIPK1‐mediated necroptosis accompanied by HMGM1 release [142]. All of these studies indicate that the response of cancer cells to PDT by necroptosis is highly dependent on the dose of the PS and the energy of the applied light.

### Immunogenic necroptosis in PDT

Recent comprehensive in vitro and in vivo studies provide evidence that PDT-induced necroptosis can be immunogenic. For instance, PDT based on BAM-SiPc, an unsymmetric bisamino silicon(IV) phthalocyanine, effectively kills colon carcinoma CT26 cells by immunogenic necroptosis [143–145]. Notably, besides the release of several DAMPs from dying cancer cells (e.g., ATP, HMGB1, CRT, ERp57, HSP90 and vasostatin) the suppression of CD47 (the “don’t eat me” signal) and up-regulation of inflammatory chemokines (CXCL1–3, 10, 12 and 13) have also been observed [143]. All these are required for the development of an effective immune response, as well as phenotypic maturation of DCs characterized by increased expression of CD80^+^, CD86^+^ and MHCII^+^ and increased production of IL-12 and IFN-γ [144]. BAM-SiPc-based vascular PDT leads to activation of anti-tumor immunity in a T-cell-dependent manner. This immunity contributed to the eradication of tumors in 70% of tumor-bearing Balb/c mice and protected against re-challenge with tumor cells for over a year [145]. In addition, the increase of IL-4 and IL-10 levels suggest that the humoral immune response was involved in the development of anti-tumor immunity [145].

It is interesting that PDT based on a novel PS, tetrakis[4-(4-fluorobenzyoxy)] phenyl-tetracyanoporphyrazine, induced a mixed types cell death (apoptosis and necroptosis) and triggered a pronounced immunogenic response in vitro (e.g., release of ATP and HMGB1, activation of BMDCs) and demonstrated the protective effect against fibrosarcoma MCA205 cells in the tumor prophylactic vaccination model in immunocompetent C57BL/6 J mice [15]. Notably, in that study tumor growth in the vaccinated immunodeficient BALB/c Nude mice was intense, which points to a significant role of the adaptive immune system in protecting against tumor growth. All of these studies indicate that induction of necroptosis seems to be a promising strategy for boosting PDT anticancer therapy by activating the antitumor immune response and overcoming tumor resistance to apoptosis.

### PDT-induced necroptosis in nano-theranostics

In addition to the development of new effective strategies for anti-cancer therapy, an equally important issue is the timely diagnosis of the tumor. Recently, nano-theranostic agents intended for MRI-guided photothermal/PDT (PTT/PDT) have been developed. The advantage of this approach is simultaneous monitoring by MRI imaging and killing of tumors with high specificity and therapeutic efficiency by induction of PTT/PDT without causing systemic side effects [16, 146]. It is noteworthy that cancer cells triggered by MRI-guided PTT/PDT undergo necroptosis. The dominant role of necroptosis was observed in the treatment of human ovarian carcinoma A2780 cells using CuS–MnS_2_ nano-flowers [146]. The cancer cell death was accompanied by increased phosphorylation of MLKL, while the apoptosis pathway was inhibited (expression of cleaved caspase-3 ↓, Bcl2/Bax2 ratio↑) and anti-apoptosis cell signaling pathways were activated (PI3K↓, AKT↑, ERK↑) [146]. Moreover, triggering both mitochondria-mediated apoptosis and necroptosis by modulating the MLKL/Actin-Capping Protein (CAPG) pathway has been shown in MRI-guided PTT/PDT therapy of human gastric carcinoma based on CuS–NiS_2_ nanomaterial [16]. However, the immunogenicity of tumor cell death induced by these nanotheranostic agents has not been studied. The development of such technologies in the future will enable not only effective tumor treatment but also early-stage screening for tumors to achieve timely diagnosis and adjustment of the anti-cancer therapy.

## Ferroptosis

### A brief overview of ferroptosis

In 2012, the research group of B. Stockwell discovered a new cell death modality selectively triggered by the oncogenic RAS-selective lethal small molecule, erastin. This cell death modality was named ferroptosis (Fig. 2) [147, 148]. The term “ferroptosis” is derived from the Greek word “ptosis” (falling) and the Latin word “ferrum” (iron). Ferroptosis is a form of regulated necrotic cell death associated with iron-dependent oxidative modification of phospholipid membranes. The unique morphological, biochemical and genetic characteristics of ferroptosis make it possible to distinguish it from the conventional types of cell death. Several inhibitors of ferroptosis have been identified, including ferrostatin-1, the inhibitor of ROS and lipid peroxidation, liproxstatin-1, and the iron chelator, deferoxamine [130].

Morphologically, ferroptosis is characterized by shrinkage of mitochondria with increased membrane density, reduction/disappearance of mitochondrial cristae, and outer mitochondrial membrane rupture [77, 148]. The nucleus size remains unchanged, and neither chromatin condensation nor margination is observed [148–151]. Recent atomic force microscopy studies in murine fibrosarcoma L929 cells revealed that the development of ferroptosis is accompanied by cellular shrinkage and appearance of uniform circular protrusions of the plasma membrane accompanied by a gradual decrease in its elasticity [131]. Despite the formation of surface roughness in the early stages of ferroptosis, the сytoskeleton remains intact and membrane blebs can be distinguished [131].

Ferroptosis is regulated by multiple genes primarily related to iron homeostasis and lipid peroxidation metabolism (e.g., GPX4, TFR1, SLC7A11, NRF2, NCOA4, P53, HSPB1, ACSL4, FSP1), which have been reviewed recently [77, 152, 153]. However, the race to find the novel specific ferroptosis regulator genes is ongoing. Biochemically, ferroptosis was initially characterized by its association with blockade of the cystine/glutamate antiporter (system xc) or suppression of glutathione peroxidase 4 (GPX4). Normally, the activity of system xc^−^ (heterodimer composed of SLC7A11 and SLC3A2 subunits) provides a cystine supply and is responsible for maintaining a cellular antioxidant environment that prevents ROS production. GPX4 is a selenoprotein required for detoxifying lipid peroxides and decreasing the activity of phospholipid peroxidation inducers. Disrupting the system xc^-^ or decreasing the levels of GPX4 leads to exhaustion of antioxidant cysteine–glutathione (GSH) metabolism. In this regard, lipid peroxides cannot be metabolized, and Fe^2+^ oxidizes lipids *via* the Fenton reaction, which leads to the production of highly toxic lipid peroxides and cell death [154, 155]. Therefore, a complex interplay between lipid, iron and cysteine metabolism is an important factor in the regulation of ferroptotic processes [77]. Lipid peroxides are considered an essential marker of ferroptosis: the higher the level of lipid peroxides inside the cell, the more aggressive the ferroptosis [156].

Ferroptosis inducers other than erastin are known, including the small molecule compound Ras Selective Lethal 3 (RSL3) (inactivates GPX4) [149], FIN56 (ferroptosis-inducing 56, promotes the degradation of GPX4 and reduces the abundance of antioxidant CoQ10) [157], FINO_2_ (indirectly inhibits GPX4 enzymatic function and directly oxidizes iron, causing widespread lipid peroxidation) [158, 159].

### Immunogenicity of ferroptosis and its role in cancer treatment

The evidence so far indicates that ferroptosis may be involved in diverse biological processes in mammals as well as the emergence and further development of several pathological processes, such as ischemia‒reperfusion injury, renal failure, nervous system diseases, hematologic diseases, and tumorigenesis [153, 160]. Ferroptosis has also been shown to be a potential therapeutic strategy for melanoma, colorectal cancer, hepatocellular carcinoma, and cancers of the pancreas, stomach, breast, lung, and head and neck [77, 152]. Recent studies have shown that high GPX4 expression levels and low HMOX1 expression levels are poor prognostic factors for patients with esophageal squamous cell carcinoma [161].

From the viewpoint of anticancer therapy, ferroptosis is considered as an alternative cell death mode for eradicating tumors resistant to apoptosis and/or necroptosis. It is noteworthy that the process of ferroptosis could enhance the immune response because the danger signals released from ferroptotic cells may act as adjuvants for stimulating the immune system. Recently, it was shown that early, but not late, ferroptotic cancer cells release classical DAMPs (e.g., ATP, HMGB1) with subsequent phenotypic maturation of BMDCs [32]. The authors demonstrated effective vaccination in immune-competent mice but not in immune-compromised Rag-2^−/−^ mice, suggesting that the mechanism of ferroptosis immunogenicity is very tightly regulated by the adaptive immune system and is dependent on the cell death stage [32]. The established immunogenic potential of ferroptotic cell death opens new possibilities for the development of therapy against poorly treated tumors, including metastatic tumors.

Since iron plays a pivotal role in ferroptosis, novel strategies are focused on the construction of iron-based nanomaterials, for example, ferumoxytol [162], inorganic iron nanoparticles [163], and iron-organic frameworks [164], with the aim of sensitizing the cells to ferroptosis. A recently designed biomimetic magnetic nanoparticle (Fe_3_O_4_-SAS@PLT) built from sulfasalazine (SAS)-loaded mesoporous magnetic nanoparticles (Fe_3_O_4_) and platelet (PLT) membrane camouflage effectively triggered ferroptosis by inhibiting system xc^−^ and improving the efficacy of programmed cell death 1 (PD-1) immune checkpoint blockade therapy in a mouse model of metastatic tumors of murine breast cancer 4T1 [165]. That study showed that Fe_3_O_4_-SAS@PLT repolarizes macrophages from the immunosuppressive M2 phenotype to the antitumor M1 phenotype, which effectively inhibits metastatic tumor growth. All of these studies indicate that ferroptosis can be immunogenic. Development of novel cancer immunotherapy based on ferroptosis induction is a possibility.

### Ferroptosis: PDT and nano-theranostics

Evidence has emerged in the last few years that PDT can also induce ferroptosis in tumor cells. For example, 5-ALA induces ferroptosis *via* regulation of GPX4 and heme oxygenase 1 (HMOX1) and exerts antitumor effects in esophageal squamous cell carcinoma cell lines and BALB/cAJcl-nu/nu mice with subcutaneously transplanted KYSE30 cells [161].

Studies on ferroptosis induction by PDT are closely linked with the design of third-generation PSs: nanocomplexes/nanocontainers combining PSs with pharmacological agents, PSs incorporated into liposomes, PSs conjugated with sugar molecules, monoclonal antibodies, and peptides [11]. The development of nano-constructs is advantageous because it helps to simultaneously solve several issues that limit PDT efficacy. First, such nano-complexes target the delivery of PSs and pharmacological agents to cancer cells. The use of PSs that absorb in the near infra-red spectrum (NIR, 600–1000 nm) allows light to penetrate deep into the tumor tissue. This enables the reduction of the PS dose without losing its effectiveness, and thus significantly diminishes cytotoxicity to normal cells. The inclusion of additional pharmacological agents in such platforms increases the induction rate of cell death and leads to the activation of the anti-tumor immune responses needed for tumor eradication and prevention of future metastasis.

Moreover, the development of nano-platforms provides an opportunity for facilitating photodynamic reactions. For instance, to overcome the hypoxia-related limitation of PDT, oxygen-boosted PDT uses agents that increase oxygen concentration in the tumor microenvironment [11]. Providing the tumor tissue with the oxygen required for ROS generation during PDT significantly increases the effectiveness of therapy for solid tumors. At the same time, the observed recruitment of T lymphocytes to the tumor site in oxygen-boosted PDT contributes to increasing the secretion of IFN-γ, which in turn downregulates SLC3A2 and SLC7A11 and strengthens ferroptosis [166, 167].

The development of multi-modal nano-platforms also enables synergistic photothermal/photodynamic/chemodynamic cancer therapies, which have excellent anti-tumor effects, and a stronger immune response that controls the growth of both the primary and distant (untreated) tumors, as reported for murine breast cancer 4T1 in vitro and in vivo [168]. It has also been reported that nano-constructs can contain the PS alone or combined with ferroptosis-inducing drugs, and in both cases they lead to induction of ferroptosis [33, 169].

Nevertheless, despite the above-mentioned advantages, the developers of nano-constructs for ferroptosis induction face several challenges, including maintaining a balance between excessive iron-induced toxicity and insufficient iron delivery. Thus, during the development of nano-constructs, one should take into account both their biocompatibility with normal and tumor cells and their ability to provide unimpeded development of ferroptosis and maintenance of the efficiency of photodynamic reactions [170, 171].

### Combining ferroptosis with other cell death modalities – a new PDT perspective

Recent studies indicate that PDT can trigger ferroptosis together with other cell death modalities. This is especially relevant for highly resistant tumors consisting of actively proliferating cells and dormant cells that enter reversible G0 cell cycle arrest, thereby posing a risk of metastases and evasion of immunological control [172]. Since the rapidly proliferating cells are sensitive to apoptosis and the dormant cells are sensitive to ferroptosis, induction of mixed cell death modalities might be useful for dealing with tumor resistance and preventing cancer recurrence [33, 173].

Recently developed is a photosensitizing cyclometalated Ir^III^ complex derived from benzothiophenylisoquinoline with imidazophenanthroline and its derivative as an ancillary ligand (IrL1 and MitoIrL2), together with a mitochondria-targeting triphenylphosphonium group to activate apoptosis [17]. Both constructs can induce ferroptosis, but the use of MitoIrL2 provides stronger synergy between ferroptosis and apoptosis. In this setup, greater PDT efficacy is achieved against refractory cancer cell lines such as triple-negative MDA-MB-231 cells and apoptosis-resistant PANC-1 cells under hypoxia and in the model of 3D multicellular spheroids of MCF-7 cells. This approach opens new perspectives for combating hypoxic and apoptosis-resistant tumor cells.

Induction of mixed cell death types has also been shown in another approach based on PDT and methylene blue [174, 175]. This approach was effective against highly aggressive triple-negative breast cancer MDA-MB-231 cells; it induced lysosomal membrane permeabilization, which led to ferroptosis and necroptosis. However, in the MCF-7 cell line, a model of the less aggressive luminal A subtype of breast cancer, which lacks significant amounts of oxidizable phospholipids and thus lacks lipid peroxidation, ferroptosis did not contribute to the death of PDT-induced cells [174, 175].

Interestingly, photosens-based PDT can activate both apoptosis and ferroptosis in murine glioma GL261 cells, and the cell death has pronounced immunogenic properties [14]. Dying cancer cells emit DAMPs such as CRT, HMGB1 and ATP, which are efficiently engulfed by BMDCs. The BMDCs then mature, become activated, and produce IL-6; they demonstrated effective vaccination potential in the mouse tumor prophylactic vaccination model using fibrosarcoma MCA205 cells.

Recently published studies describing the immunogenic nature of ferroptosis support the idea that ferroptosis induction by PDT can be a powerful alternative strategy for increasing the efficacy of cancer therapy. Further insights into the interplay and synergism between PDT and ferroptosis, as well as the possibility of inducing mixed cell death modalities (ferroptosis with apoptosis and/or ferroptosis with necroptosis) may provide new possibilities for the development of novel cancer immunotherapy against resistant and highly malignant tumors.

#### Escape mechanisms of tumor cells from PDT

Сell-fate decisions elicited by PDT are largely dependent on the photochemical properties and biological effects of the PS, its concentration, the irradiation dose and, more importantly, on the genetic, epigenetic and phenotypic tumor profiles. High tumor heterogeneity even between tumors of the same origin and sub-classification can be one of the main causes of a large discrepancy between the responses of tumor cells to PS uptake and PDT-induced activation of cell death modalities on the one hand, and the development of tumor resistance to the therapy on the other hand. In this regard, a recent review by Aniogo and colleagues described in detail the possible molecular pathways that might be responsible for the escape of tumor cells from PDT [176]. Among the main mechanisms preventing the cytotoxic effects of PDT, the following elements might play an important role: (1) altered genetic profile, and particularly altered expression of tumor survival genes [177–179], (2) increase in DNA damage-repair processes [180], (3) increase in AKT/mTOR signaling, (4) expression of ROS-scavenger proteins (e.g., glutathione S-transferase (GST), glutathione peroxidase 4 (GPx4)) [181, 182], and generation of nitric oxide (NO) [183, 184]. Moreover, PDT can cause dysregulation of the antiapoptotic Bcl-2 protein at the mitochondrial membrane, overexpression of efflux proteins (e.g., P-glycoproteins), and formation of autolysosomes through recycling of cytoplasmic content for cell survival [176, 185]. Moreover, resistance of tumor cells to PDT can also develop due to dysfunctional apoptosis [176, 186]. On the one hand, activation of autophagy can attenuate the development of apoptosis and other cell death modalities; on the other hand, the pro-death roles of autophagy can lead to autophagic cell death, especially in cells with defective apoptosis [176, 187]. Considering the specific tumor cell profile, different protocols use PSs with different physicochemical properties and at different concentrations, as well as a variety of irradiation doses. So, it is extremely difficult to predict in advance the exact type of cell death triggered in a particular patient. and this has generated extensive scientific debate. Therefore, detailed characterization of tumor cells and their degree of sensitivity to PDT is urgently needed. Several in vitro and in vivo approaches for isolation and selection of PDT-resistant tumor cells are known [188, 189]. These models can be used for detailed study of cell morphology and population characteristics, molecular mechanisms of sensitivity to PSs (based on inherent and induced resistance in different cell lines), and metastatic abilities of tumor cells in mice. Such approaches could lay the ground for establishing strategies for induction of alternative regulated forms of cell death, preferably with hallmarks of ICD capable of overcoming resistance to PDT.

An equally important challenge is the development of tumor-specific markers to predict tumor sensitivity to PDT in order to select patients for PDT. Generic markers for prediction of PDT sensitivity are needed, as well as markers highly specific for each tumor type.

Recent discussions at the Annual Congress of the European Society for Photodynamic Therapy (Euro-PDT) in 2020 pointed out potential markers of the efficiency of PDT-based methyl aminolaevulinic acid cream (MAL-PDT) in patients with basal cell carcinoma. These markers were immunopositivity to p53 (good patient responsiveness to PDT) and increased immunostaining of β-catenin, the expression of which is linked to tumor aggressiveness (poor patient responsiveness to PDT) [190]. In patients suffering from Bowen’s disease, positive p53 immunostaining correlated with good response to PDT [190]. Good response of patients with actinic keratoses to daylight PDT was associated with hypofunction of the PIK3R1 gene [190]. Based on experimental results and data from patients with cholangiocarcinoma, Zhang and colleagues built a prognostic model to identify four ferroptosis-related genes (SLC2A1, SLC2A6, SLC7A5 and ZEB1) that can be considered as targets for PDT sensitivity or resistance to ferroptosis [191]. All these findings indicate the importance of identifying predictive clinical markers for assessing the feasibility and efficacy of PDT that could lead to PDT-personalized therapy. Therefore, these studies should not be neglected but should be intensified. Many new challenging studies in this direction are expected, which will help to clarify the criteria for cancer sensitivity to PDT.

### Concluding remarks and future perspectives

Clinical use of PDT began in the middle of the 20th century and has taken its place among the therapeutic tools for different types of cancer. However, since cancer remains a serious challenge for public health, there is a need to review and revise the current approaches for boosting the efficacy of tumor eradication and eliminating the risk of tumor recurrence and metastasis while maintaining the quality of patients’ lives.

**Fig. 3:**
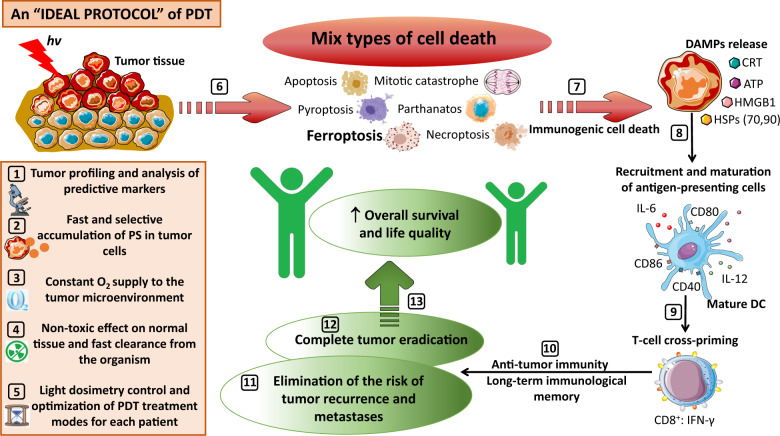
An “ideal” protocol for PDT. An ideal protocol for PDT should comply with the following requirements. [1] Tumor profiling and analysis of predictive markers for evaluation of the degree of tumor sensitivity to PDT. [2] The PS should have a constant chemical composition and photochemical characteristics that enable it to rapidly and selectively accumulate in tumor cells and to gain a strong cytotoxic effect during its photoinduction. [3] The approach should provide at least a sufficient oxygen supply to the tumor microenvironment for successful generation of photodynamic reactions. [4] The concentration of the PS and the irradiation dose should be tailored to the tumor’s origin and have minimal toxic side effects on normal tissue, even in the absence of photoinduction. [5] The PDT protocol should include a light dosimetry control procedure for timely optimization of PDT treatment modes for each patient. [6] Cancer cells exposed to PDT can proceed through several cell death pathways with either immunogenic or non-immunogenic properties that determine the therapeutic outcome of PDT according to the different scenarios. In an ideal protocol, the PS and treatment modes should lead to the simultaneous induction of several regulated forms of immunogenic cell death. We suggest that PDT-induced ferroptosis could be combined with other cell death modalities to boost PDT efficacy. Finally, PDT should activate immunogenic cell death pathways [7] accompanied by the release of damage-associated molecular patterns (DAMPs) such as ATP, HMGB1 and HSP, and by CRT exposure on the outer cell surface in order to trigger the recruitment and maturation of antigen-presenting cells (e.g., DCs). [8] This will result in optimal antigen presentation to CD8^+^ T cells [9], induction of antitumor immunity, and generation of long-lasting immunological memory [10]. The immune system response is successfully engaged in the suppression of tumor growth [11] and will help to deal with any remaining tumor cells, including distant metastatic cells [12]. Therefore, activation of immunogenic cell death modalities plays a crucial role in the therapeutic success of PDT and hence the overall survival and life quality of patients [13].

Looking ahead, the ideal protocol for PDT should comply with the following requirements (Fig. 3). First and most important is characterization of tumor profiles and analysis of predictive markers for assessment of the degree of tumor sensitivity to PDT for selection of patients eligible for PDT. Second, is the choice of a PS with constant chemical composition and photochemical characteristics. It should rapidly and selectively accumulate in tumor cells and effectively trigger cell death during photoinduction. Next, the concentration of PS and irradiation dose should be tailored to the tumor’s origin and have minimal toxic effects on normal tissue, even in the absence of photoinduction. In addition, the approach should provide an oxygen supply to the tumor microenvironment to guarantee successful photodynamic reactions. Finally, PDT should activate immunogenic cell death pathways, enable the immune system to deal with any remaining tumor cells, including distant metastatic cells, and generate a long-lasting immunological memory.

In recent years, it has become clear that cancer cells exposed to PDT can proceed through several different cell death pathways that have either immunogenic or non-immunogenic properties. Which pathway(s) they go through determines the outcome of PDT. The variety of cell death modes make it possible to avoid cancer cell resistance to certain types of death, and particularly apoptosis and necroptosis. Following the novel concept of personalized medicine, it has become evident that PDT approaches should also focus on providing light doses based on the characteristics of the individual tumors. The use of a specific PS and combined with a specific PDT power will lead to the induction of alternative regulated forms of cell death with hallmarks of ICD. It is important to emphasize that PDT enables the use of combinations of the same PS with different irradiation regimens to trigger different cell death responses. This could be the fundamental basis for the development of PDT regimens, which should be focused on simultaneous induction of several cell death modalities, increasing treatment efficacy, and preventing the acquisition of resistance to cancer therapy. Though modern PSs can penetrate well into tumors, the most intense photodynamic reactions proceed on the tumor surface due to the limited penetration of light, which means that the activity of photodynamic reactions in cells depends on the location of the cells in the tumor. Therefore, the PDT procedure must take into account that low irradiation intensities in deep tumor layers can have a pro-tumorigenic effect resulting in the development of tumor resistance to the treatment. The use of photodynamic agents that induce regulated cell death modalities at both high and low light intensities is an attractive solution. Importantly, the cell death types in the deep layers of the tumor could differ from those on the surface, but it is highly desirable that the ICD modalities will be induced. Combining ferroptosis induction by PDT with induction of other cell death modalities could boost the efficacy of the treatment. It has been proposed that since both PDT and ferroptosis rely on the Fenton reaction, which ultimately leads to the production of lipid ROS, they can work synergistically and possibly achieve a stronger induction of cancer cell death [78]. Further insights into the interplay and synergism between PDT and ferroptosis, as well as the possibility of combining ferroptosis with other cell death modalities, may provide new ground for the development of novel cancer immunotherapy against resistant and highly malignant tumors. Indeed, this is a challenging research area and many novel research studies are expected.

## Data Availability

All relevant data are included in this manuscript.
